# Safety and Usefulness of Cryobiopsy and Stamp Cytology for the Diagnosis of Peripheral Pulmonary Lesions

**DOI:** 10.3390/cancers11030410

**Published:** 2019-03-22

**Authors:** Tatsuya Imabayashi, Junji Uchino, Akihiro Yoshimura, Yusuke Chihara, Nobuyo Tamiya, Yoshiko Kaneko, Tadaaki Yamada, Koichi Takayama

**Affiliations:** Department of Pulmonary Medicine, Graduate School of Medical Science, Kyoto Prefectural University of Medicine, Kyoto 602-8566, Japan; imabayas@koto.kpu-m.ac.jp (T.I.); aki-y@koto.kpu-m.ac.jp (A.Y.); c1981311@koto.kpu-m.ac.jp (Y.C.); koma@koto.kpu-m.ac.jp (N.T.); kaneko-y@koto.kpu-m.ac.jp (Y.K.); tayamada@koto.kpu-m.ac.jp (T.Y.); takayama@koto.kpu-m.ac.jp (K.T.)

**Keywords:** cryobiopsy, bronchoscopy, cell biology, ultrasonography, lung cancer

## Abstract

Reports on the use of cryobiopsy (CB) for lung cancer diagnosis are limited. The aims of the present study were to evaluate the safety and usefulness of CB using radial endobronchial ultrasonography, without a guide sheath, for the diagnosis of peripheral pulmonary lesions and determine the utility of stamp cytology, an on-site diagnostic technique for determining tumor inclusion in CB samples. We retrospectively analyzed data for 35 patients (36 lesions) with suspected peripheral lung cancer who underwent CB between August 2017 and February 2019 at our medical facility. The diagnostic yield, incidence of complications, and the utility of stamp cytology for diagnosis were investigated. The diagnostic yield of CB was 86.1% (31/36) with histological diagnosis, and 80.5% (29/36) with diagnosis using stamp cytology; the overall yield was 91.6% (33/36). Pneumothorax requiring thoracic drainage occurred in two patients, both of whom had lesions contacting the pleura. Grade 2 and grade 1 bleeding occurred in one and 25 patients, respectively. CB enables the collection of very large, nearly intact tissue samples, thus resulting in an improvement in the true diagnosis rate and facilitating the measurement of multiple biomarkers as well as rapid histological diagnosis.

## 1. Introduction

Lung cancer is associated with a very high mortality rate, with non-small cell lung cancer (NSCLC) accounting for 85% of cases [1]. For end-stage NSCLC, along with the promotion of techniques based on individualized medicine, the list of immunohistochemical and molecular tests necessary for determining treatment plans and objectives is increasing on a daily basis. Of late, the development of BRAF (v-raf sarcoma viral oncogene homolog B1 V600E) screening via next-generation sequencing (NGS) [2,3,4] has resulted in the need for newly frozen cell samples instead of formalin-fixed, paraffin-embedded ones. Unfortunately, the conventional technique of forceps biopsy (FB) does not allow the collection of sufficient, good-quality cells, a problem that continues to become more common.

The attachment of a flexible cryoprobe to bronchoscopes was first reported in a study that examined its usefulness for eliminating tracheobronchial obstruction [5]. Since the latter half of the 20th century, cryobiopsy (CB) has been used for the diagnosis of diffuse lung diseases [6,7,8] and central lung cancers [9,10,11,12,13,14]. This technique enables the collection of much larger and more or less intact samples than FB. Accordingly, we anticipated that it would be useful for the immunohistochemical and molecular analysis of peripheral pulmonary lesions (PPLs), which are common in NSCLC. However, reports on the use and safety of this technique for the diagnosis of PPLs are few [15,16,17,18]. Furthermore, the collection of multiple samples via CB, which is not possible with FB, can present high risks and may be technologically difficult.

The aims of the present study were to investigate the usefulness and safety of CB using radial endobronchial ultrasonography (EBUS), without a guide sheath (GS), for the diagnosis of PPLs and to evaluate the utility of stamp cytology, an on-site diagnostic technique for determining tumor inclusion in CB samples.

## 2. Experimental Section

### 2.1. Subjects

**Figure 1:**
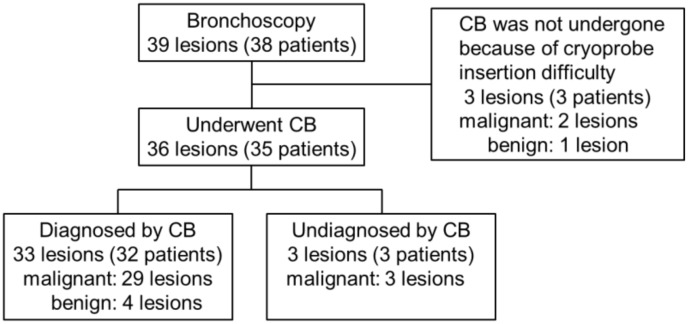
Flow diagram showing the results of cryobiopsy (CB) in 35 patients with peripheral pulmonary lesions.

We retrospectively analyzed data for patients with suspected peripheral lung cancer who underwent CB procedures at our facility between August 2017 and February 2019 (Figure 1). This retrospective study was approved by the Ethics Review Board of Kyoto Prefectural Medical School Hospital (ERB-C-1190).

### 2.2. Bronchoscopy and Biopsy Procedures

Bronchoscopy was performed under local anesthesia with 30–50 μg fentanyl and 2 mg midazolam for sedation. All cases were successfully intubated, and a 1T-Q290 (distal end diameter, 5.9 mm; working channel (WC) diameter, 3.0 mm) or P290 (distal end diameter, 4.1 mm; WC diameter, 2.0 mm) scope (Olympus, Tokyo, Japan) was used. EBUS procedures were performed using an endoscopic ultrasound device (EU-ME1; Olympus, Tokyo, Japan) and a 1.4-mm radial EBUS probe (UM-S20–17S; Olympus, Tokyo, Japan). Before bronchoscopy, a virtual bronchoscopic pathway indicating the bronchial route to the lesion was prepared from helical computed tomography (CT) data (1.0-mm slice width) by using a virtual bronchoscopic navigation (VBN) system (Bf-NAVI^®^; Cybernet Systems, Tokyo, Japan). For cryosurgery, the ERBECRYO^®^ 2 was used with a cryoprobe with a diameter of 1.9 mm (ERBE, Tubingen, Germany), with carbon dioxide as the cooling agent. 

**Figure 2:**
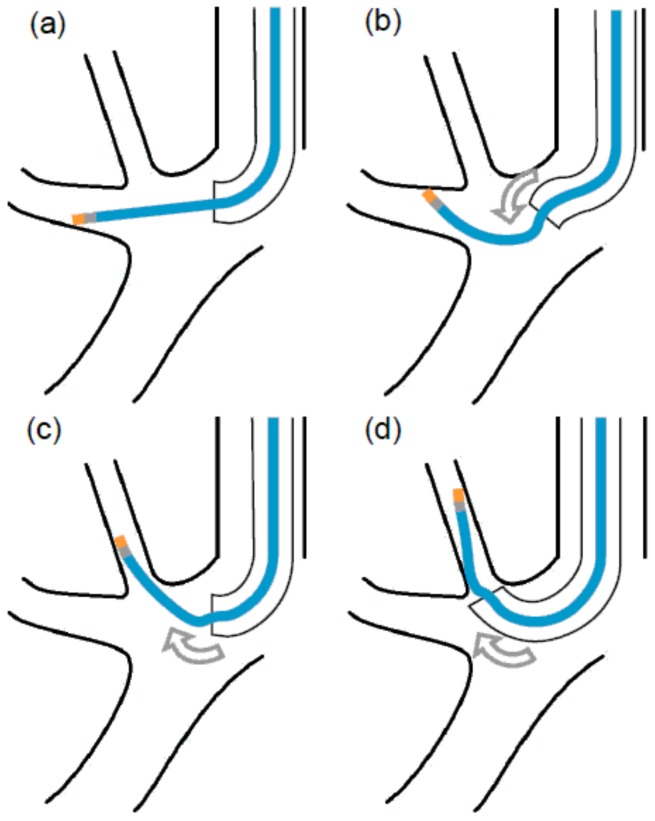
The cryoprobe bending method for peripheral pulmonary lesions. (**a**) When the cryoprobe has been bent as far as possible toward the lung apex, (**b**) the scope is bent in a down angle. (**c**) The shape memory property of the probe can be used to push it completely toward the lung apex. (**d**) Forward feeding of the cryoprobe instead of a guidewire, with simultaneous advancement of the scope, enables the confirmation of probe entry into the correct bronchus.

For access to a region with no strong flexion, such as the right middle lobe, one should follow the VBN guide, advance the scope as close as possible to the lesion, insert a direct EBUS probe into WC, and visualize it under fluoroscopy. If the access to the lesion can be confirmed on the EBUS screen, the optimal location of the lesion for biopsy can be identified by fluoroscopy. The EBUS probe should be navigated between the tip of the scope and the lesion two-to-three times in order to ensure that access to the lesion is reproducible. If the lesion is near the pleura, the identification site should be at least 1 cm away from the pleura. After the EBUS probe is removed and the scope is fixed, a cryoprobe is inserted and the biopsy is performed under fluoroscopic guidance. However, when a cryoprobe is inserted into the apex of the lung, which shows strong flexion, the metal tip can get caught and refuse to pass along the U-bend of the scope. This phenomenon may occur when GS is used. Therefore, we used a cryoprobe bending method without GS in the present study. After identifying the biopsy site using the EBUS probe and fluoroscopic guidance, the biopsy site is returned to the carina and the cryoprobe tip is inserted into the position visible on the endoscope. In this situation, the up angle of the scope is approximately 100°, and insertion into apical branch of right upper lobe bronchus is normally quite difficult. We used the shape memory property of the cryoprobe; bending it by pressing it against the bronchial wall enabled us to successfully push the probe up to the lung apex. (Figure 2). Bleeding was prevented by using a 5- or 7-Fr Arndt^®^ endobronchial blocker (Cook Medical, Bloomington, IN, USA). In February 2018, the blocker was discontinued in the Japanese market, and it became difficult to use it in all cases. We placed the blocker in the lower lobe and other easily accessible regions and found coarse vascular echoes in the vicinity of the tumor echo on the EBUS screen. We also placed it in the center of the lesion, where the risk of hemorrhage was high. When blocker placement was difficult, the freezing time was changed to 2 s.

The CB tissue samples were stamped onto two glass slides and either fixed in formalin or frozen. One slide was spray-fixed and treated with the Papanicolaou stain, while the other was dry-fixed and stained with Diff-Quik. 

Brushing was also performed prior to CB in all cases and prior to FB in some cases. Rapid on-site evaluation of brushing was performed in some cases, and FB was not performed if the diagnosis was confirmed. A chest radiograph was routinely obtained to identify pneumothorax 2 h after the procedures.

### 2.3. Diagnosis

Cases of CB with or without concurrent FB and brushing, with no specific findings other than inflammatory changes, were included in this study if the shadows disappeared on subsequent follow-up CT.

### 2.4. Bleeding

Several methods to grade the severity of bleeding have been reported in the literature [9,10,11,12,13,14]. However, argon plasma coagulation cannot be easily performed for bleeding from PPLs. Moreover, we needed to ensure uniform grading, regardless of the use of an endobronchial blocker. Accordingly, grades were assigned according to the following criteria: grade 0, no bleeding or extremely minimal bleeding that does not require suction; grade 1, mild bleeding that can be controlled by suction or 3 min of pressure via an inflated balloon catheter [19]; grade 2, moderate bleeding with blood flowing over the inserted tracheal tube (bleeding amount, >30 mL [13]) that requires more than 3 min of pressure via an inflated balloon catheter; and grade 3, severe bleeding requiring transfusion, general care, and/or surgery.

### 2.5. Statistical Analysis

Descriptive statistics were used to analyze patient characteristics. Normally distributed continuous data are described as means ± standard deviations (ranges), while categorical variables are reported as percentages of the total number of subjects. Several continuous and categorical variables were analyzed using the Kruskal–Wallis test. All statistical analyses were performed using Easy R for Windows, version 1.35 (Saitama Medical Center, Jichi Medical University, Saitama, Japan). A *p*-value of <0.05 was considered statistically significant.

## 3. Results

Three patients could not undergo cryoprobe insertion because of strong flexion of the lesion site (LB1+2a or RB6a). These patients were excluded, and data for a total of 35 patients with 36 lesions, including 15 men and 20 women with a mean age of 66.9 ± 10.3 years (range: 44–81 years) were retrospectively analyzed. An endobronchial blocker was used for prophylactic hemostasis in seven patients. The mean lesion size was 37.2 ± 19.4 mm (range: 8–84 mm), and the lesion locations included the upper lobe (*n* = 19), middle lobe (*n* = 2), and lower lobe (*n* = 15). The locations of the lesion relative to the hilum were as follows: inner third, *n* = 3; middle third, *n* = 21; outer third, *n* = 12. The lesions were in contact with the pleura in 11 patients. A final diagnosis of malignant and benign lesions was made for 32 and four lesions, respectively. The mean number of CBs was 1.5 ± 0.6 (range: 1–3), and the mean freezing time was 3.3 ± 0.7 s (range: 2–5 s). The EBUS probe was located within the lesion in 91.6% (33/36) cases and adjacent to the lesion in 8.3% (3/36) cases.

### 3.1. Diagnostic Yield

The diagnostic yield of CB was 86.1% (31/36) with histological diagnosis and 80.5% (29/36) with diagnosis using stamp cytology. The total yield was 91.6% (33/36), which is considerably higher than that with either procedure alone (Table 1). Three cases of malignancy in which a definite diagnosis was not obtained by CB were diagnosed by simultaneous FB and brushing. The diagnostic yield of FB and brushing was 82.7% (24/29) and 80.5% (29/36), respectively.

### 3.2. Procedure Time

The total CB procedure time, including the duration of the EBUS procedure for lesion detection, was 12.8 ± 9.2 min (range: 3–46 min). The procedure times for CB with and without endobronchial blockers were 24.2 ± 10.2 (range: 15–46 min) and 10.1 ± 6.4 (range: 3–28 min) min, respectively, while the number of CBs was 1.8 ± 0.6 (range: 1–3) and 1.4 ± 0.6 (range: 1–3), respectively (*p* < 0.001, 0.150). Thus, the time required was significantly lesser when endobronchial blockers were not used.

### 3.3. Sample Area

**Figure 3:**
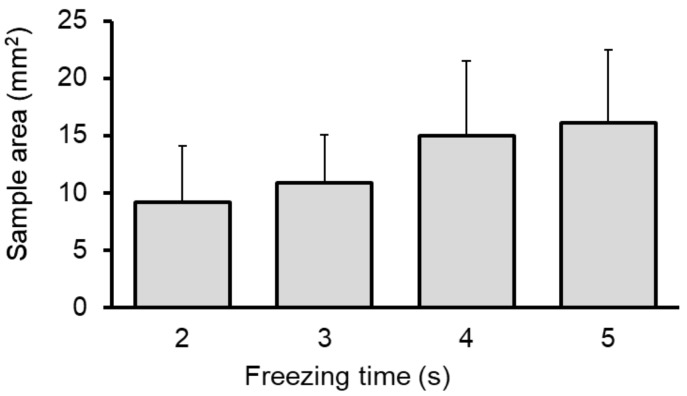
Surface area of peripheral lung lesion samples obtained via cryobiopsy according to the freezing time during cryobiopsy. The graph shows that a larger surface area can be obtained with an increased freezing time (*p* = 0.007).

The surface area of hematoxylin–eosin-stained samples, as measured by a microscope digital camera, was 12.2 ± 5.6 mm^2^ (range: 4.1–26.5 mm^2^). Figure 3 shows the sample surface area according to the freezing time during CB. The obtained surface area increased with an increase in the freezing time; it was 9.1 ± 4.6, 10.8 ± 4.1, 14.9 ± 6.3, and 16.1 ± 5.4 mm^2^ with freezing times of 2, 3, 4, and 5 s, respectively (*p* = 0.007).

### 3.4. Immunohistochemical and Molecular Analyses

Immunohistochemical and molecular tests were possible for all 26 patients diagnosed with end-stage NSCLC via CB. One of these patients showed negative findings in histological examination and positive findings in stamp cytology, which detected a G719 mutation in exon 18 of the epidermal growth factor receptor gene.

### 3.5. Complications

**Figure 4:**
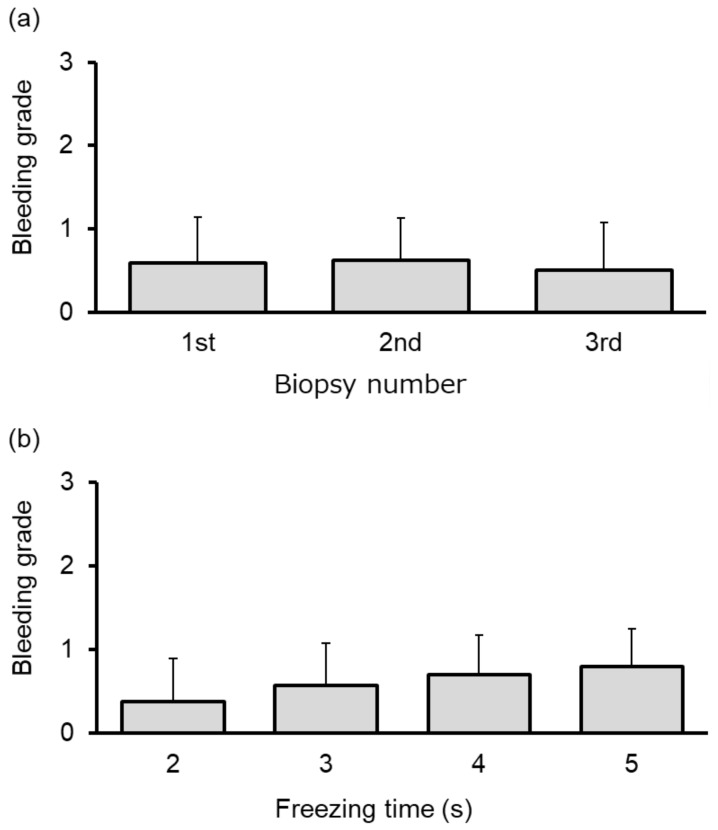
Severity (grade) of bleeding according to (**a**) the cryobiopsy number (first, second, third) and (**b**) the freezing time during cryobiopsy (2-, 3-, 4- and 5-s) in patients with peripheral pulmonary lesions. The severity of bleeding did not differ according to the number of biopsies (*p* = 0.913; Figure 4a) or the freezing time (*p* = 0.451; Figure 4b).

Pneumothorax requiring thoracic drainage occurred in two of the 35 (5.7%) patients, both of whom exhibited lesions contacting the pleura. Grade 2 and grade 1 bleeding occurred in one and 25 patients, respectively. The severity of bleeding did not differ according to the number of biopsies (*p* = 0.913; Figure 4a) or the freezing time (*p* = 0.451; Figure 4b). Pneumonia did not occur in any patient, while one patient developed mild anemia due to prolonged hemoptysis.

## 4. Discussion

In the present study, we demonstrated the usefulness and safety of CB for the diagnosis of PPLs.

The biggest limitation of CB from PPLs is the difficulty in access caused by the rigidity of the cryoprobe. In particular, the procedure is exceedingly difficult in strongly curved regions of the lung, such as the lung apex. In the present study, FB was not performed for all patients, the biopsy number was not specified beforehand, and the analysis was retrospective and limited to a single center; therefore, it was difficult to compare the results for CB with those for FB in order to evaluate the diagnostic power. However, patient tolerability was better with FB than with CB in terms of the approach and high-risk lesions, such as those contacting the pleura or large blood vessels. If cryoprobes with a diameter smaller than the currently used diameter of 1.9 mm [15,20] enter the market, access to difficult regions should become easier. On the other hand, the large size of samples obtained by CB is a distinct advantage when the EBUS probe is adjacent to the lesion, as observed in cases of submucosal or external wall lesions [8]. However, only thinly sliced portions can be pathologically examined, which increases the possibility of missed diagnosis. In the present study, two patients showed negative findings in the histological examination and positive findings in stamp cytology, which can prevent missed diagnosis. While it is known that stamp cytology can improve the diagnostic yield of FB [21], we speculated that the technique could be similarly applied to CB and would be quite useful for confirming tumor inclusion. Finally, because CB samples are much larger than FB samples, crushing damage caused by the stamp cytology technique is no longer a concern.

Bleeding, a worrisome complication, was only slight when CB was performed under the EBUS guidance in order to avoid large blood vessels [22]. With regard to the correlation between the freezing time during CB and the severity of bleeding, the freezing time was not randomized in our study. A 2 s freezing time was selected in nearly all cases where bleeding was moderate or where we were unable to completely avoid major blood vessels despite the EBUS guidance. The incidence of bleeding for the 2 s cases was 37.5% (3/8), whereas that for the 3 s, 4 s and 5 s cases was 57.1% (16/27), 70.5% (12/17) and 80.0% (4/5), respectively. As opposed to diffuse lung diseases, lung cancer does not require large tissues for diagnosis, therefore, a decreased freezing time is an effective technique when bleeding is a concern. With regard to the correlation between the number of CBs and bleeding severity, second and third procedures were required for 44.4% (16/36) and 11.1% (4/36) patients, respectively. Because the total number of biopsies performed in these cases was increased, the overall bleeding rate was higher. However, severe bleeding was not observed in any case [14]. 

Because indwelling manipulation of endobronchial blockers is easy in the right middle and bilateral lower lobes, the procedure is a good adaptation. However, in highly stenotic bronchi, the blocker interferes with the cryoprobe and is consequently difficult to place. In addition, the indwelling manipulation of the blocker is complicated when it is placed in the upper lobe, and there is a possibility of loosening after biopsy. As a result, the device was not used in all patients in our study. Moreover, despite the fact that our cases were slightly polarized, the examination time tended to be longer for patients with complications than for those without. 

Other than bleeding, pneumothorax occurred as a complication of CB in our study. Both patients with pneumothorax exhibited lesions adjoining the pleura; this necessitated the use of fluoroscopy to maintain a large enough margin between the lesion and biopsy site.

## 5. Conclusions

In conclusion, CB can exhibit a high diagnostic yield and is associated with a low incidence of bleeding in cases of PPLs where CB and simultaneous EBUS are possible. Furthermore, the use of stamp cytology with CB not only facilitates the on-site confirmation of tumor inclusion and an improved diagnostic yield, but also lowers risks by ensuring that the number of biopsies is kept to a minimum. However, the complication risk and patient tolerability should be thoroughly evaluated. In addition, clinicians need to master the technique via hands-on practice or other means. Further large-scale, randomized, prospective, comparative trials are necessary to further confirm our findings.

## Figures and Tables

**Table 1:** Diagnostic yield of cryobiopsy according to the size, location, and diagnosis of peripheral pulmonary lesions.

Variables	Diagnostic Yield of Cryobiopsy					
	Histology		Stamp Cytology		Total	
Lesion size						
≤30 mm	82.3	(14/17)	76.4	(13/17)	88.2	(15/17)
>30 mm	89.4	(17/19)	84.2	(16/19)	94.7	(18/19)
Location of the lobe						
Right upper lobe	87.5	(14/16)	81.2	(13/16)	87.5	(14/16)
Right middle lobe	50.0	(1/2)	100	(2/2)	100	(2/2)
Right lower lobe	83.3	(5/6)	66.6	(4/6)	100	(6/6)
Left upper lobe	100	(3/3)	66.6	(2/3)	100	(3/3)
Left lower lobe	88.8	(8/9)	88.8	(8/9)	88.8	(8/9)
Location relative to the hilum						
Central	66.6	(2/3)	66.6	(2/3)	100	(3/3)
Intermediate	90.4	(19/21)	80.9	(17/21)	95.2	(20/21)
Peripheral	83.3	(10/12)	83.3	(10/12)	83.3	(10/12)
Location of the EBUS ^1^ probe						
Within the lesion	90.9	(30/33)	78.7	(26/33)	90.9	(30/33)
Adjacent to the lesion	33.3	(1/3)	100	(3/3)	100	(3/3)
Final diagnosis						
Malignant	84.3	(27/32)	81.2	(26/32)	90.6	(29/32)
Adenocarcinoma	88.8	(16/18)	88.8	(16/18)	94.4	(17/18)
Squamous cell carcinoma	75.0	(3/4)	50.0	(2/4)	75.0	(3/4)
Small cell carcinoma	75.0	(3/4)	75.0	(3/4)	75.0	(3/4)
Adenosquamous carcinoma	100	(1/1)	100	(1/1)	100	(1/1)
Large cell carcinoma	100	(1/1)	100	(1/1)	100	(1/1)
Carcinoid	100	(1/1)	0	(0/1)	100	(1/1)
Metastatic carcinoma	66.6	(2/3)	100	(3/3)	100	(3/3)
Benign	100	(4/4)	75.0	(3/4)	100	(4/4)
IgG4 related lung disease	100	(1/1)	0	(0/1)	100	(1/1)
Granuloma	100	(1/1)	100	(1/1)	100	(1/1)
Inflammatory change	100	(2/2)	100	(2/2)	100	(2/2)
Total	86.1	(31/36)	80.5	(29/36)	91.6	(33/36)

Data are presented as % (*n*/*N*). ^1^ endobronchial ultrasonography.

## References

[1] Hecht S.S. (2003). Tobacco carcinogens, their biomarkers and tobacco-induced cancer. Nat. Rev. Cancer.

[2] Planchard D., Kim T.M., Mazieres J., Quoix E., Riely G., Barlesi F., Souquet P.J., Smit E.F., Groen H.J., Kelly R.J. (2016). Dabrafenib in patients with BRAF(V600E)-positive advanced non-small-cell lung cancer: A single-arm, multicentre, open-label, phase 2 trial. Lancet Oncol..

[3] Planchard D., Besse B., Groen H.J.M., Souquet P.J., Quoix E., Baik C.S., Barlesi F., Kim T.M., Mazieres J., Novello S. (2016). Dabrafenib plus trametinib in patients with previously treated BRAF(V600E)-mutant metastatic non-small cell lung cancer: An open-label, multicentre phase 2 trial. Lancet Oncol..

[4] Planchard D., Smit E.F., Groen H.J.M., Mazieres J., Besse B., Helland Å., Giannone V., D’Amelio A.M., Zhang P., Mookerjee B. (2017). Dabrafenib plus trametinib in patients with previously untreated BRAF^V600E^-mutant metastatic non-small-cell lung cancer: An open-label, phase 2 trial. Lancet Oncol..

[5] Mathur P.N., Wolf K.M., Busk M.F., Briete W.M., Datzman M. (1996). Fiberoptic bronchoscopic cryotherapy in the management of tracheobronchial obstruction. Chest.

[6] Ganganah O., Guo S.L., Chiniah M., Li Y.S. (2016). Efficacy and safety of cryobiopsy versus forceps biopsy for interstitial lung diseases and lung tumours: A systematic review and meta-analysis. Respirology.

[7] Lentz R.J., Argento A.C., Colby T.V., Rickman O.B., Maldonado F. (2017). Transbronchial cryobiopsy for diffuse parenchymal lung disease: A state-of-the-art review of procedural techniques, current evidence, and future challenges. J. Thorac. Dis..

[8] Hetzel J., Maldonado F., Ravaglia C., Wells A.U., Colby T.V., Tomassetti S., Ryu J.H., Fruchter O., Piciucchi S., Dubini A. (2018). Transbronchial Cryobiopsies for the Diagnosis of Diffuse Parenchymal Lung Diseases: Expert Statement from the Cryobiopsy Working Group on Safety and Utility and a Call for Standardization of the Procedure. Respiration.

[9] Schumann C., Hetzel J., Babiak A.J., Merk T., Wibmer T., Moller P., Lepper P.M., Hetzel M. (2010). Cryoprobe biopsy increases the diagnostic yield in endobronchial tumor lesions. J. Thorac. Cardiovasc. Surg..

[10] Aktas Z., Gunay E., Hoca N.T., Yilmaz A., Demirag F., Gunay S., Sipit T., Kurt E.B. (2010). Endobronchial cryobiopsy or forceps biopsy for lung cancer diagnosis. Ann. Thorac. Med..

[11] Hetzel J., Eberhardt R., Herth F.J.F., Petermann C., Reichle G., Freitag L., Dobbertin I., Franke K.J., Stanzel F., Beyer T. (2012). Cryobiopsy increases the diagnostic yield of endobronchial biopsy: A multicentre trial. Eur. Respir. J..

[12] Jabari H., Sami R., Fakhri M., Kiani A. (2012). Different protocols for cryobiopsy versus forceps biopsy in diagnosis of patients with endobronchial tumors. Pneumologia.

[13] Rubio E.R., Le S.R., Whatley R.E., Boyd M.B. (2013). Cryobiopsy: Should this be used in place of endobronchial forceps biopsies?. Biomed. Res. Int..

[14] Segmen F., Aktas Z., Özturk A., Kizilgöz D., Yilmaz A., Alici I.O., Demirağ F., Pehlivanoğlu P. (2017). How many samples would be optimal for endobronchial cryobiopsy?. Surg. Endosc..

[15] Schuhmann M., Bostanci K., Bugalho A., Warth A., Schnabel P.A., Herth F.J., Eberhardt R. (2014). Endobronchial ultrasound-guided cryobiopsies in peripheral pulmonary lesions: A feasibility study. Eur. Respir. J..

[16] Herath S., Yap E. (2017). Novel hybrid cryo-radial method: An emerging alternative to CT-guided biopsy in suspected lung cancer. A prospective case series and description of technique. Respirol. Case Rep..

[17] Arimura K., Tagaya E., Akagawa H., Nagashima Y., Shimizu S., Atsumi Y., Sato A., Kanzaki M., Kondo M., Takeyama K. (2018). Cryobiopsy with endobronchial ultrasonography using a guide sheath for peripheral pulmonary lesions and DNA analysis by next generation sequencing and rapid on-site evaluation. Respir. Investig..

[18] Taton O., Bondue B., Gevenois P.A., Remmelink M., Leduc D. (2018). Diagnostic Yield of Combined Pulmonary Cryobiopsies and Electromagnetic Navigation in Small Pulmonary Nodules. Pulm. Med..

[19] Echevarria-Uraga J.J., Pèerez-Izquierdo J., Garcìa-Garai N., Gòmez-Jiménez E., Aramburu-Ojembarrena A., Tena-Tudanca L., Miguélez-Vidales J.L., Capelastequi-Saiz A. (2016). Usefulness of an angioplasty balloon as selective bronchial blockade device after transbronchial cryobiopsy. Respirology.

[20] Yarmus L.B., Semaan R.W., Arias S.A., Feller-Kopman D., Ortiz R., Bösmüller H., Illei P.B., Frimpong B.O., Oakjones-Burgess K., Lee H.J. (2016). A Randomized Controlled Trial of a Novel Sheath Cryoprobe for Bronchoscopic Lung Biopsy in a Porcine Model. Chest.

[21] Kawaraya M., Gemba K., Ueoka H., Nishii K., Kiura K., Kodani T., Tabata M., Shibayama T., Kitajima T., Tanimoto M. (2003). Evaluation of various cytological examinations by bronchoscopy in the diagnosis of peripheral lung cancer. Br. J. Cancer.

[22] Berim I.G., Saeed A.I., Awab A., Highley A., Colanta A., Chaudry F. (2017). Radial Probe Ultrasound-Guided Cryobiopsy. J. Bronchol. Interv. Pulmonol..

